# Heterogeneity of CD4^+^CD25^+^Foxp3^+^Treg TCR β CDR3 Repertoire Based on the Differences of Symbiotic Microorganisms in the Gut of Mice

**DOI:** 10.3389/fcell.2020.576445

**Published:** 2020-09-01

**Authors:** Jun Li, Huaijuan Xue, Qingqing Ma, Xiaoyan He, Long Ma, Bin Shi, Suhong Sun, Xinsheng Yao

**Affiliations:** ^1^Department of Immunology, Center of Immunomolecular Engineering, Innovation & Practice Base for Graduate Students Education, Zunyi Medical University, Zunyi, China; ^2^Department of Laboratory Medicine, Guizhou Aerospace Hospital, Zunyi, China; ^3^Department of Breast Surgery, The Affiliated Hospital of Zunyi Medical University, Zunyi, China

**Keywords:** gut microbes, intestinal mucosal immune, Treg, TCR, CDR3 repertoire

## Abstract

Gut microbes play a crucial role in the occurrence and development of autoimmune diseases. The diversity of intestinal microorganisms affected by the living environment, regulate the immune function of peripheral immune organs and local tissues. In the study, the diversity of intestinal microorganisms of Germ-free (GF), Specific Pathogen-free (SPF), and Clean (CL) BALB/c mice were conducted by 16S rDNA sequencing. High-throughput sequencing technology was used to analysis the composition and characterization of TCR β chain CDR3 repertoires in Regulatory T cells (Treg) in intestine and spleen of GF, SPF, and CL mice, so as to investigate the effects of differential composition of intestinal microorganisms on the CD4^+^CD25^+^Foxp3^+^Treg TCR β CDR3 repertoire of intestine and spleen. We observed that GF, SPF, and CL mice have different gut microorganism composition, and the abundance and quantity of microorganisms are positively correlated with the level of feeding environment. Interestingly, incomplete structure of spleen and small intestine in GF mice was found. Moreover, a significant difference in the usage of high frequency unique CDR3 amino acid sequences was detected in the intestinal Treg TCRβ CDR3 repertoire among GF, SPF and CL mice, and there were a greater heterogeneity in the usage frequency of *TRBV, TRBJ*, and *TRBV-TRBJ* combinations gene segments. However, the effect of different feeding environment on the mice Treg TCRβ CDR3 repertoire of spleen was weak, implying that the different composition of intestinal microbiota may primarily affect the diversity of the local Treg TCRβ CDR3 repertoire and does not alter the overall properties of the circulating immune system. These results provide basic data to further analyze the mechanism of gut microbes regulating the intestinal mucosal immune system.

## Introduction

The colonization of gut microbes is not only very important for the metabolism of essential nutrients, but also plays an essential role in the development of the immune system (Rooks and Garrett, 2016). Bacteria and symbiotic microflora in the gut can dynamically regulate the occurrence and development of autoimmune diseases (Ivanov et al., 2009; Brown et al., 2011; Lee et al., 2011; Scher et al., 2013; Cantarel et al., 2015). Through natural selection and coevolution with the host, some microbial groups form an inseparable host microorganism symbiosis relationship with host in physiology and pathology (Sommer and Bäckhed, 2013). Symbiotic microflora affects the formation of immune system by participating in innate and adaptive immunity as well as the development and maintenance of various supervision mechanisms.

The diversity of symbiotic bacteria is different in mice raised in different environments, such as feed, drinking water and living environment, which has an important impact on the occurrence and development of their peripheral immune system. It was found that the development of peripheral immune organs of mice in aseptic feeding environment was significantly disordered compared with that of SPF mice (Telesford et al., 2015). However, transfer the intestinal microorganisms of SPF mice in GF mice, this defect was obviously improved (Macpherson and Harris, 2004; Mazmanian et al., 2005), indicated that the gut flora had an important regulatory effect on the development of peripheral immune system (Ivanov and Honda, 2012; Wu and Wu, 2012). Moreover, Ivanov et al. (2008) found that the differentiation of Th17 cells in the lamina propria of the small intestine required specific symbiotic microflora, which can regulate the balance between Foxp3 + Treg cells and Th17 cells in the lamina propria of the small intestine, suggesting that the composition of intestinal microflora is the key point of coordination (Agace and McCoy, 2017).

High throughput sequencing technology has the characteristics of accuracy, high efficiency and sensitivity, which has been widely used in the sequence analysis of T / B cell repertoire (Setliff et al., 2019; Roskin et al., 2020). At present, the knowledge is lack of influence on the CDR3 sequence of β chain of Treg cells in peripheral immune organs (spleen) and local tissues (intestine) caused by gut microbes. In this study, 16S rDNA sequencing and High-throughput sequencing were applied to detect the gut microbes composition and CD4^+^CD25^+^Foxp3^+^Treg repertoire of spleen and intestine in GF, SPF, and CL mice. The aim of this study was to investigate the relationship between gut microbe composition and CD4^+^CD25^+^Foxp3^+^Treg repertoire.

## Materials and Methods

### Material and Sample Collection

A total of nine female BALB/c mice were purchased from the Department of experimental zoology, the basic Department of the China Third Military Medical University. Among them, there were three Germ-free (GF) mice, Specific Pathogen-free (SPF) mice and Clean (CL) mice, respectively, each weighing 21–24 g. GF, SPF, and CL mice were raised strictly according to the standard. All animals and experiments were conducted according to the guidance of animal care and use of laboratory animals (Ministry of Health, China) and approved by the experimental animal and use Ethics Committee of Zunyi Medical University. Feces of all mice were collected and sent to Beijing Genomics Institution (BGI) for sequencing of intestinal microorganisms. Spleens, intestine and mesenteric lymph nodes were collected at the time of sacrifice.

### Morphological Analysis

The spleen, intestine and mesenteric lymph nodes were fixed in 4% paraformaldehyde overnight at 4°C, embedded in paraffin, sectioned into 8-μm thick slices, deparaffinized, and stained with hematoxylin and eosin (H&E). Tissue morphology was observed under a light microscope.

### Sequencing of Intestinal Microorganisms

Fecal DNA was extracted as described previously (Nishijima et al., 2016). 16S rDNA obtained from the fecal extraction was analyzed by Illumina sequencing according to the 16S Metagenomic Sequencing Library Preparation (15044223 B) protocol. The experimental method of 16S rDNA sequencing was described by Watanabe et al. (2020). The DNA library was sequenced using MiSeq Reagent Kit V3 (Illumina Inc.) in the MiSeq platform according to the manufacturer’s instructions. The 16S rDNA sequences were analyzed by the Quantitative Insights into Microbial Ecology (QIIME) pipeline version 1.9.1 (Caporaso et al., 2010). Through quality control, data filter out low-quality sequences, splice the sequences into tags through similarity relationship between sequences, gather tags into operational taxonomic units (OTU) based on whether they had 97% homology with the UCLUST algorithm, and then compare OTU with database (August 2013 version) to annotate species of OTU. Taxonomy from the phylum to the genus level was also performed by using the QIIME pipeline to analysis these sequences. GF mice are defined as having no detectable bacteria, fungi, protozoa and parasites in their bodies. However, one GF mouse has a certain number of gene sequencing results after PCR amplification, which may be caused by some dead microorganisms in the food after high pressure sterilization. The 16S rDNA sequences have been uploaded to NCBI repository^1^.

### CD4^+^CD25^+^Foxp3^+^Treg Cells Isolation and RNA Extraction

Single cell suspensions were prepared by grinding the tissues with the plunger of a 5 mL disposable syringe. CD4^+^CD25^+^Foxp3^+^Treg cells were obtained as described previously (Zhang et al., 2015). Briefly, CD4^+^CD25^+^Treg cells were enriched by depletion of non- CD4^+^T cells, flow-through fraction of CD4^+^T cells and positive selection of CD4^+^CD25^+^ cells according to the manufacture’s instruction of CD4^+^CD25^+^ Regulatory T Cell Isolation Kit (Miltenyi, Bergisch Gladbach, Germany). For the staining of Foxp3, cells were incubated with Cy-chrome-labeled anti-CD4 and FITC-labeled anti-CD25 monoclonal antibody. Then, according to the instructions provided by the company (eBioscience, San Diego, CA, United States), the cells were fixed and permeabilization with Intracellular Fixation Buffer after washing, stained with anti mouse Foxp3 monoclonal antibody. Finally, the purity of CD4^+^CD25^+^Foxp3^+^Treg cells were detected by flow cytometry. Genomic DNA was extracted from CD4^+^CD25^+^Foxp3^+^Treg cells using the QIAamp DNA Mini Kit (Qiagen, Milan, Italy) according to the manufacture’s instruction.

### TCR β Chain Library Preparation and High-Throughput Sequencing

The concentration and purity of genomic DNA of samples were conformed for TCR CDR3 sequencing^2^. And then genomic DNA were sent to Adaptive Biotechnologies Corp (Seattle, WA, United States) for sequencing. TCR β sequences were generated as our described previously (Ma et al., 2016). Briefly, a multiplex PCR amplification was performed consisting of 36 forward V segments (TRBV) and 14 reverse J segment primers (TRBJ) that targeted all possible somatic combinations of the rearranged TCR beta chain CDR3. Then, the TCR β CDR3 PCR library was loaded on an Illumina Flow Cell for sequencing on an Illumina Genome Analyzer (2 × 250 bp). Unfortunately, one of the samples failed to meet the requirements of database construction. The possible reason is that the DNA of SPF3-SP sample was degraded due to various factors, such as time and temperature during transportation, so it is impossible to conduct high-throughput sequencing through quality inspection.

### Bioinformatics Analysis of TCR β-Chain Library

Raw sequences in the FASTA format were processed by Immuno-SEQ analyzer toolset and IMGT/High V-QUEST (version 1.3.1) to remove the No results and Un-known sequences as well as out of frame sequences. Using Immuno-SEQ analyzer toolset and IMGT/High V-QUEST, the characteristics of the TCR beta chain CDR3 repertoire sequences were defined, including CDR3 nucleotide, CDR3 amino acid; count (reads); frequency count (%); CDR3 length; V gene name; D gene name; J gene name; V deletion; n1 insertion; D 5′ deletion; D 3′ deletion; n2 insertion; J deletion; V index; n1 index; D index; n2 index; J index; sequence status (Has stop/in frame/out frame). TCR repertoire diversity was assessed by the Anti-Simpson index. The V-J and V-D-J rearrangement of the CDR3 repertoire, the proportion and frequency of unique CDR3 sequences, CDR3 repertoire clonality, CDR3 ANIMO ACID length, CDR3 amino acid usage, V deletion and J deletion, and dominant TRBV-TRBJ combination gene segments were also calculated in different mouse class and different tissue samples. Additionally, R package “ggplot2,” “Venn Diagram,” and GraphPad Prism (version 5) were used to plot the figures. Data analysis was performed by R studio (v3.3.3) and GraphPad Prism (version 5) software. *P* < 0.05 was considered statistically significant.

## Results and Discussion

### Sequencing of Gut Microorganisms

The gut microbial genome of BALB / c mice was obtained by 16S rDNA sequencing. All SPF and CL samples passed the quality inspection and established the database. For GF samples, only one passed the quality inspection. A detailed description of the total number of raw reads and filtered reads of each sample were displayed in Additional file 1. An average of 44,780 clean reads were obtained from each individual.

Germ-free mice are defined as no detectable bacteria, fungi, protozoa and parasites (GiLbert and NeufeLd, 2014) provides an irreplaceable sterile experimental model in the study of chronic gastrointestinal diseases (Grover and Kashyap, 2014), obesity (Gérard, 2017), spontaneous arthritis (Rehaume et al., 2014), and type I diabetes (Markle et al., 2013). In this experiment, a certain amount of gene sequences of gut microorganism of GF grade mice after PCR amplification may be caused by some dead microorganisms left in the feed after autoclave, the sequencing results met the definition of GF grade mice (Rodriguez-Palacios et al., 2018).

### Species Diversity and Classification of Gut Microorganisms

**FIGURE 1:**
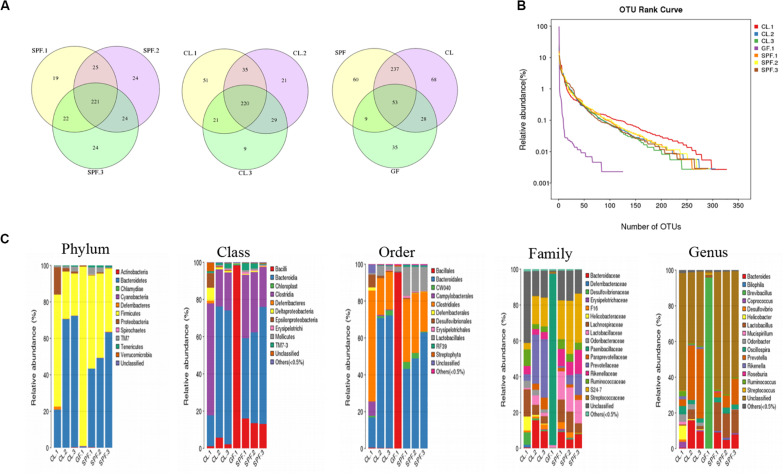
Diversity and classification of intestinal microorganisms in three different feeding environments of mice. **(A)** Venn diagram analysis the total and unique OTU number of each sample; **(B)** OTU rank curve analysis of OTU species diversity among different mice; **(C)** Taxonomic analysis of fecal diversity in BALB / c mice. GF, Germ-free class mice; SPF, Specific Pathogen-free mice; CL, Clean Class mice.

The total and unique OTU number of each sample were showed in Figure 1A according to Edgar RC (Edgar, 2013). The largest number of OTUs was found in CL mice, followed by SPF mice. All mice shared 53 common OTU. In addition, the diversity of gut microorganism among different mice were analyzed by OTU rank curve (Figure 1B). The gut microbes of CL mice were the richest not only in quantity but also in species.

Compare with the database, OTUs were classified and the proportion of each sample species was analyzed in five classification levels, including phylum, class, order, family, genus and species (Figure 1C). The species classification of gut microorganisms in the GF mice was the lowest among the five levels, while the species classification of SPF and CL mice were significantly increased by compared with GF mice. Moreover, there was difference between the dominant species of SPF and CL mice in the five-species classification, and the diversity of gut microorganisms in CL mice was the most abundant.

Symbiotic microorganisms are necessary for the occurrence and development of some clinical diseases (McMurran et al., 2019; Zhu et al., 2020). Selecting the right mouse model will be beneficial to the study of specific diseases. The above results showed that the number and species of intestinal symbiotic microorganisms were different under different feeding conditions, and the dominant microflora was also different. The difference of species classification level of gut microorganisms in mice may participate in the development of immune system of mice in three different feeding environments in a symbiotic or pathogenic way (Caricilli et al., 2014).

### Morphological Analysis of Peripheral Immune Organ

The Gut microorganism composition play an important role in the development of immune system (Martin et al., 2019). Among them, some microbial groups form an inseparable host microorganism symbiotic relationship with host physiology through natural selection and co evolution with the host (Sommer and Bäckhed, 2013). These symbiotic groups jointly affect the formation of the immune system by participating in the development and maintenance of innate and adaptive immunity and a variety of regulatory mechanisms.

**FIGURE 2:**
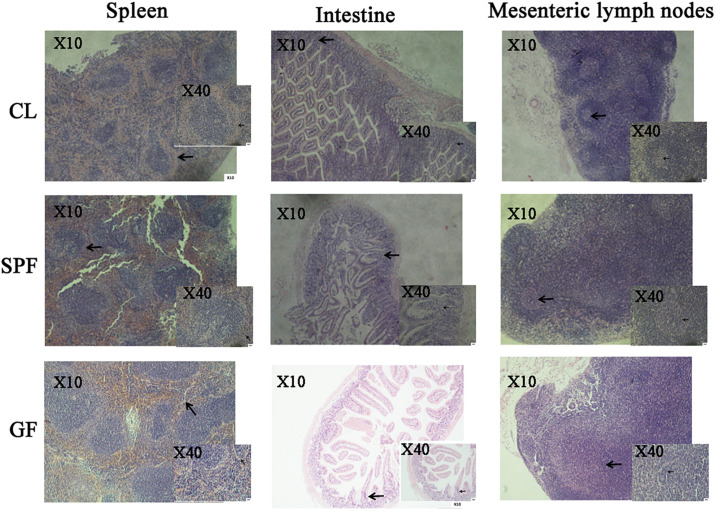
The morphology of spleen, intestine, mesenteric lymph node in different classes of BALB/c mice by H&E staining. The arrow in the spleen picture indicates the peripheral edge of the white pulp in the spleen. The arrow in the intestine picture indicates the central villi of the small intestine. The arrow in the mesenteric lymph node image indicates the lymph node. GF, Germ-free class mice; SPF, Specific Pathogen-free mice; CL, Clean Class mice.

In this study, the peripheral marginal zone of white pulp in GF mice was significantly smaller than that in CL mice by H&E staining (Figure 2). The peripheral marginal zone of white pulp contained a certain number of B and T cells (Macpherson and Harris, 2004). Therefore, we speculate that the difference of intestinal flora caused the incomplete structure of spleen, thus affecting the development of immune system in GF mice. In addition, by observing the sections of intestinal and mesenteric lymph nodes of mice of different feeding levels, we found that the angiogenesis in the center of intestinal villi of GF level mice was limited, the microvilli became longer, and the germinal center in the central area of mesenteric lymph nodes was also significantly reduced (Figure 2).

### Sorting of CD4^+^CD25^+^Foxp3^+^Treg Cells in Spleen and Intestine of Mice

Treg cells account for about 10% of the total number of peripheral T cells, which play a vital role in maintaining intestinal and peripheral tolerance and inhibiting autoimmune diseases. The expression of Foxp3 transcription factor is essential for the Treg cells development and maintenance (Hori et al., 2003; Fontenot et al., 2017). The number of CD4^+^CD25^+^Foxp3^+^Treg cells in GF mice was reported significantly reduced (Hara et al., 2013). The decrease or even disappearance of intestinal flora in GF mice may have an important effect on Treg cells.

In this study, after sorting the Treg cells in the mouse intestine and spleen, the purity of the Treg cells both in spleen and intestine reached about 80% (Additional file 2), suggesting that the purity of CD4^+^CD25^+^Foxp3^+^Treg cells can meet the requirements (Jenkins et al., 2015; Fuller et al., 2016).

### Total Sequence Data Statistics of HTS in CD4^+^CD25^+^Foxp3^+^Treg TCR β CDR3 Repertoire

CD4^+^CD25^+^Foxp3^+^Treg TCR β CDR3 repertoire were successfully constructed. A detailed information of total sequences, productive sequences, and unique sequences of each sample was displayed in Table 1. Unique sequence (Torella et al., 2014) is one of the key factors to analyze the specificity of the repertoire. The average value of unique sequences in intestine was 113, which is much lower than that in spleen.

**TABLE 1:** Total sequences statistics of TCR beta chain CDR3 repertoire.

Sample	Total	Out and Stop of frame	In frame	Productive
CL1-In	192	58	134	126
CL1-SP	16926	4904	12022	12008
CL2-In	114	50	64	59
CL2-SP	27266	7875	19391	19351
CL3-In	44	26	19	15
CL3-SP	27126	7757	19369	19340
SPF1-In	113	53	60	58
SPF1-SP	28491	8692	19799	19772
SPF2-In	60	30	30	27
SPF2-SP	58428	16718	41710	41643
SPF3-In	997	420	577	569
GF1-In	223	122	101	99
GF1-SP	27088	8635	18453	18416
GF2-In	106	58	49	40
GF2-SP	4155	1538	2617	2604
GF3-In	195	94	101	101
GF3-SP	27879	8747	19132	19089

*GF, Germ-free class mice; SPF, Specific Pathogen-free mice; CL, Clean Class mice; SP, spleen; In, intestine.*

### Clonal Expansion of Treg TCR β Chain CDR3 Repertoire

**FIGURE 3:**
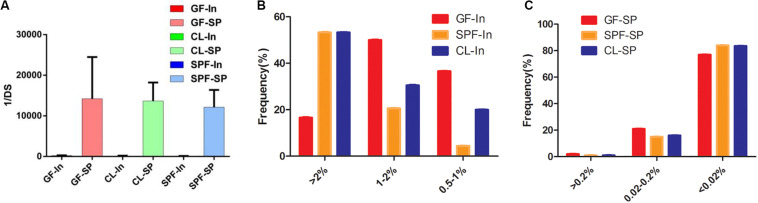
Expansive of total Treg TCR beta CDR3 clones in spleen and intestine. **(A)** The inverse Simpson’s diversity index average distribution; **(B,C)** Expansive degree in spleen and intestine. GF, Germ-free class mice; SPF, Specific Pathogen-free mice; CL, Clean Class mice. SP, spleen; In, intestine.

It is reported that the diversity of T cell receptor (TCR) can reach 2 × 10^6^ (Nishio et al., 2015) in the peripheral of mice after massive replication. The diversity of TCRs is predominantly confined to the CDR. The CDR3 domain is in direct contact with peptide antigens and is highly diverse, allowing the recognition of various antigens (Attaf et al., 2015). The Anti Simpson Index (1 / DS) was used to evaluate the diversity of Treg TCR β chain CDR3 in BALB / c mice. The results showed that the diversity of Treg TCR β chain CDR3 repertoire in spleen was significantly higher than that in intestine of all samples (Figure 3A). However, there was no statistical significance through the statistical analysis of 1 / DS average distribution in different mice class of intestine and spleen, suggesting that differences in intestinal microorganisms has no obvious effect on the diversity of Treg TCR β CDR3 of intestine and spleen.

The expansive level of Treg TCR β chain CDR3 repertoire can directly reflect the immune response of T cells and can also be used for the immune reconstruction after antiviral treatment or bone marrow transplantation, as well as the detection of autoimmune diseases, including biomarkers for the treatment of type I diabetes (Kern et al., 2014; Shevyrev and Tereshchenko, 2019). The expansive level of each clonotype in intestine (Figure 3B) were conducted, GF mice was mainly in medium frequency (1–2%), while the CL and SPF mice were mainly in high frequency (>2%). For spleen, the clone and proliferation level of Treg TCR β CDR3 repertoire in all mice were mainly in low frequency (<0.02%), and with no statistical significance in the cloning frequency (Figure 3C). It is suggested that the difference of feeding environment has no specific effect on the diversity and clonality of Treg TCR β CDR3 repertoire in the spleen of BALB / c mice.

All samples shared “YEQY” and “NTLY” amino acid motif among five sequences amplified at high frequency (Additional file 3), and the highest frequency was found in CL mice, which may be related to the stimulation of common antigen epitopes in the environment.

### Usage Frequency of *V, J* and *V-J* Combination Gene Segments in TCR β Chain CDR3 Repertoires

**FIGURE 4:**
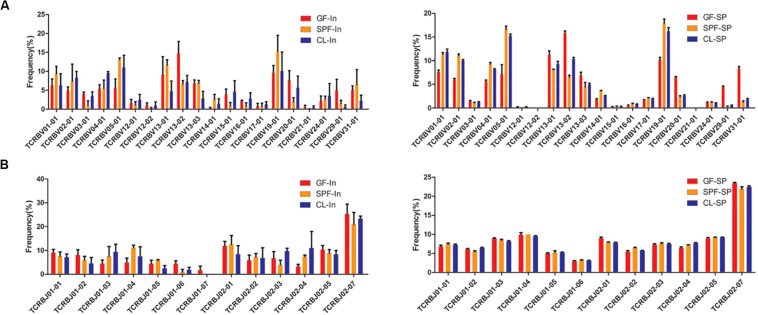
Distinct usage percentages of the total *TRBV* and *TRBJ* genes in mice spleen and intestine. **(A)** usage percentages of the TRBV; **(B)** usage percentages of the TRBJ. GF, Germ-free class mice; SPF, Specific Pathogen-free mice; CL, Clean Class mice. SP, spleen; In, intestine.

The patterns of *TRBV* and *TRBJ* gene usage were determined dominantly by MHC alleles. The same high frequency usage *TRBV* and *TRBJ* genes were detected both in spleen and intestine among all samples, such as *TRBV19-01* and *TRBJ02-07*. Moreover, the intestine of GF mice has their own unique high-frequency usage genes, such as *TRBV13-02* and *TRBJ02-01* (Figure 4). Compared with GF mice, *TRBV05-01* and *TRBV01-04* were up-regulated in intestine of CL and SPF mice. However, high frequency usage *TRBV* and *TRBJ* genes do not differ in spleen among GF, SPF, and CL mice.

To further study the characteristics of *TRBV* and *TRBJ* gene usage, the expression level of the 100 most high frequently usage *TRBV-TRBJ* combinations were compared among GF, SPF, and CL mice. There are common dominant pairing genes in spleen and intestine of all mice and different group of mice also have their own unique advantage matching genes in the intestine and spleen (Additional file 4). *TRBV19-01-TRBJ02-07* and *TRBV05-01-TRBJ02-07* were dominant combinations identified in intestine and spleen, respectively in all mice.

Between-individual similarities in usage of V, J, and V-J combinations gene segments may stem from chromatin conformation, physical proximity of germline segments, and/or recombinatorial bias (Pickman et al., 2013). Selective bias of V and J gene usage may lead to a loss of diversity in the intestinal TCR repertoires and result in occurrence of some diseases, such as inflammatory bowel disease (Saravanarajan et al., 2020). Although the species of symbiotic bacteria have changed in different environmental levels, there is still a common specific antigen. Therefore, the same high frequency of *TRBV* and *TRBJ* gene family of Treg TCR β CDR3 repertoire in the gut and spleen of mice were found, and some specific high frequency of usage may be related to the change of specific symbiotic bacteria after the change of environmental level.

### Amino Acid Analysis of TCR Beta Chain CDR3 Repertoire

**FIGURE 5:**
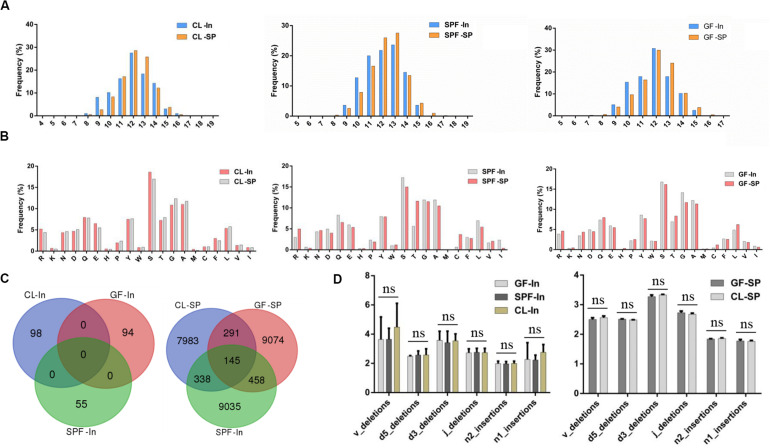
Amino acid analysis of TCR beta chain CDR3 repertoire. **(A)** Length distribution of TCR beta chain CDR3 repertoire in spleen and intestine; **(B)** Top 20 AA usage distribution of Treg TCR beta chain CDR3 repertoire in spleen and intestine; **(C)** overlap of Treg TCR beta chain CDR3 regions amino acid sequences in spleen and intestine. **(D)** Statistical analysis of the distribution of TCR-β CDR3 region nucleotides deletion and insertion in spleen and intestine. GF, Germ-free class mice; SPF, Specific Pathogen-free mice; CL, Clean Class mice. SP, spleen; In, intestine.

Different types of T cells have different length distribution of CDR3 and can combine with different MHC complexes. It has been confirmed that the CDR3 length distribution of CD4^+^ T cells is significant different in patients with IgG4-related disease (Wang et al., 2019). The distribution of amino acid length in all samples was similar, and the distribution of amino acids length was concentrated between 8 and 16 amino acids (Figure 5A). All mice formed bell-shaped distribution, which peaks at 12 amino acids, advocating that the change of intestinal flora does not affect the distribution of CDR3 amino acids sequence of Treg TCR β chain in spleen and intestine.

Moreover, hydroxy hydrophilic amino acids were taken at high frequency in the CDR3 region of Treg TCR β chain in spleen and intestine represented by serine (Figure 5B). However, no significant statistical significance was found among the samples after statistical analysis. These results suggested that the difference of environment has no specific effect on the high frequency use of amino acids in the CDR3 region of Treg TCR β chain in the gut and spleen of BALB / c mice.

The same amino acid overlapping sequence did not appear in the intestine among 3 groups but was found in the spleen (Figure 5C). The same amino acid overlapping sequence in the spleen may be the natural Treg (Cebula et al., 2013) derived from mouse thymus, which is not related to the specific antigen stimulation in the environment.

The cleavage and insertion of CDR3 region of Treg TCR β chain have been reported to be an important mechanism for the formation of CDR3 region diversity (Toor et al., 2016). However, our comparative analysis of the cleavage and insertion of CDR3 region of Treg TCR β chain in spleen and intestine of all experimental samples showed that nucleotide insertion and deletion occur at the junction of V, D, and J gene segments did not significant difference among GF, SPF, and CL mice (Figure 5D), which may be caused by sample size. Therefore, future investigations will be needed to verify the associations in the larger population.

## Conclusion

In this study, we evaluated the correlation between the differences of intestinal microflora and the heterogeneity of CD4^+^CD25^+^Foxp3^+^Treg TCR β CDR3 repertoire by analyzing the differences of intestinal microflora in different feeding environments and sequencing CD4^+^CD25^+^Foxp3^+^Treg cell in the intestine and spleen. GF, SPF, and CL mice have different intestinal microbial composition, which may be related to the development and composition of different peripheral immune tissues and organs. In addition, the significant difference of Treg TCR β CDR3 repertoire among GF, SPF, and CL were found only in intestine, indicating that different intestinal microbial composition may mainly affect the diversity of intestinal Treg TCR β CDR3 repertoire. However, the mechanism of intestinal microbes regulating intestinal immune system is still unknown, and further confirmatory investigations will be needed to conduct the effects of intestinal microorganisms and metabolites on intestinal immune cells.

## Data Availability Statement

The datasets presented in this study can be found in online repositories. The names of the repository/repositories and accession number(s) can be found in the article/Supplementary Material.

## Ethics Statement

The animal study was reviewed and approved by all animals and experiments were conducted according to the guidance of animal care and use of laboratory animals (Ministry of Health, China) and approved by the Experimental Animal and Use Ethics Committee of Zunyi Medical University.

## Author Contributions

XY designed the research. JL and HX did the experiment and wrote the manuscript. QM, XH, LM, BS, and SS analyzed parts of the data. All authors contributed to the article and approved the submitted version.

## Conflict of Interest

The authors declare that the research was conducted in the absence of any commercial or financial relationships that could be construed as a potential conflict of interest.

## Footnotes

1 https://www.ncbi.nlm.nih.gov/bioproject/PRJNA646285/

2 http://www.immunoseq.com
