# Supplementary material for: Heterogeneity of CD4+CD25+Foxp3+Treg TCR β CDR3 Repertoire Based on the Differences of Symbiotic Microorganisms in the Gut of Mice
Source: Front Cell Dev Biol. 2020 Sep 1;8:576445. doi: 10.3389/fcell.2020.576445 (PMC7490519; doi:10.3389/fcell.2020.576445)
Supplement: Supplementary file 1 [file Data_Sheet_1.docx]

**Additional file1:**

Table 1 Statistics of sequencing of intestinal microorganisms


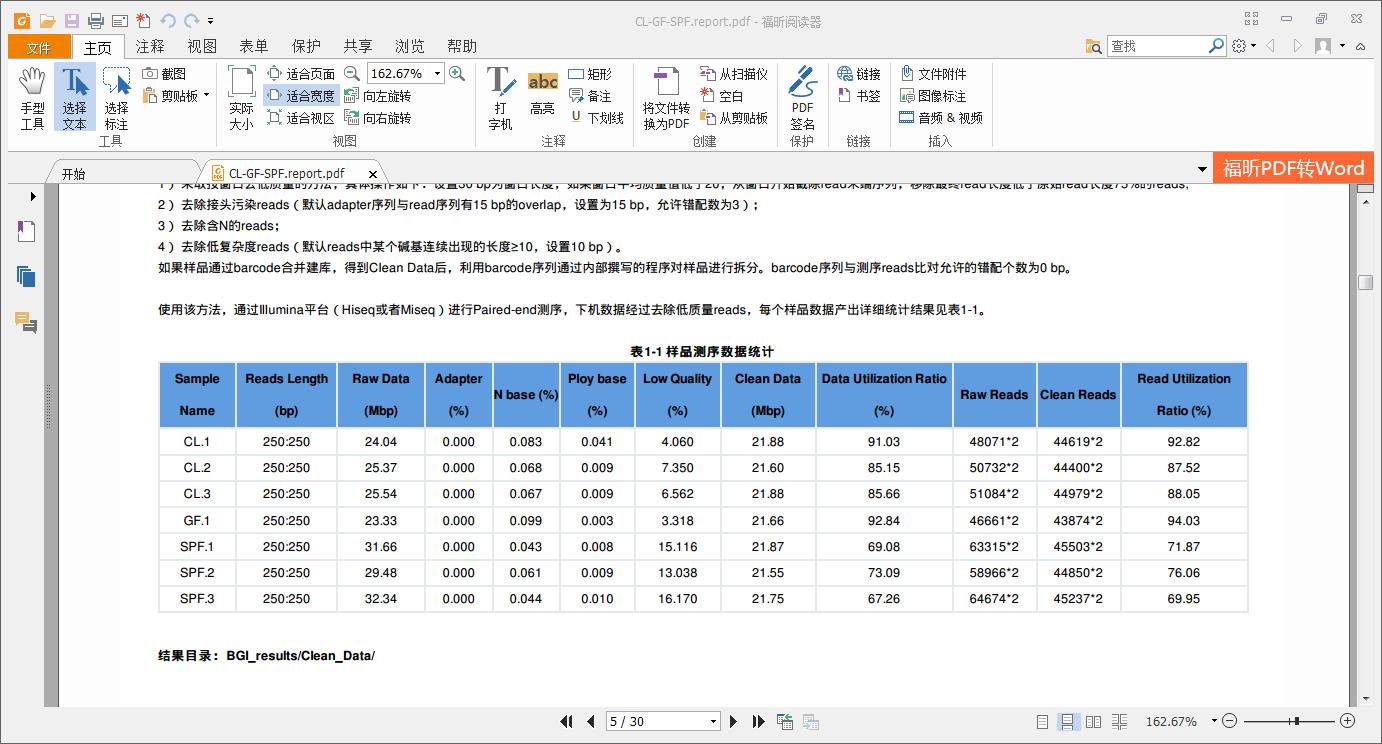


GF: Germ-free class mice; SPF: Specific Pathogen-free mice; CL: Clean Class mice.

**Additional file 2 (Figure 1-2):**


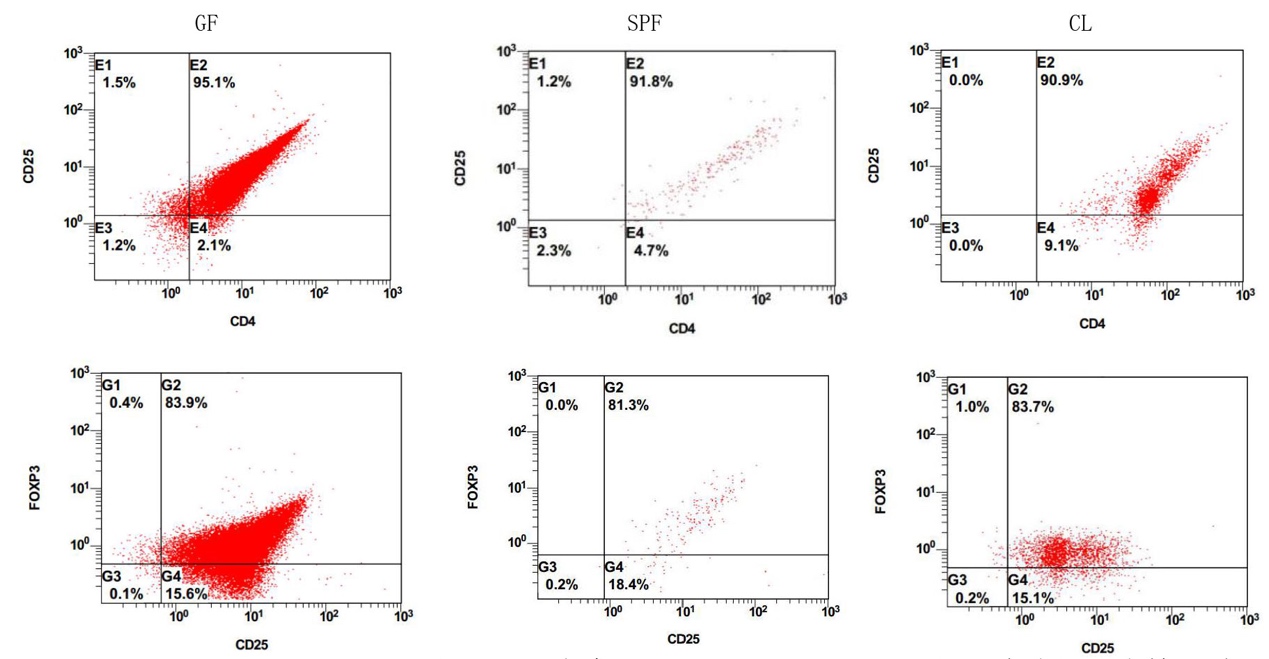


Figure 1. Flow cytometric analysis of spleen CD4+CD25+Foxp3+Treg cells in different classes of NO.3 BALB/c mice. GF: Germ-free class mice; SPF: Specific Pathogen-free mice; CL: Clean Class mice.


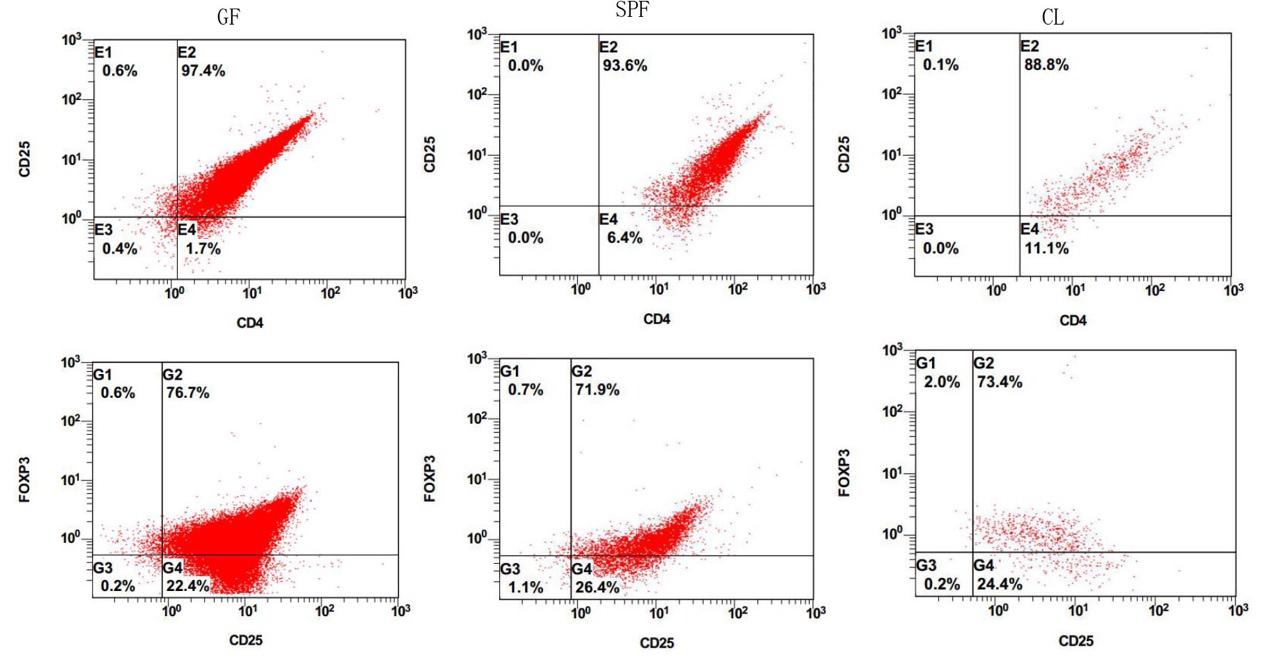


Figure 2. Flow cytometric analysis of spleen CD4+CD25+Foxp3+Treg cells in different classes of NO.3 BALB/c mice. GF: Germ-free class mice; SPF: Specific Pathogen-free mice; CL: Clean Class mice.

**Additional file 3 (Table 2-4):**

Table 2 the top five expressed CDR3 sequences from the GF group of BALB/c mice in spleen and intestine

| SampLe | V gene | J gene | CDR3 sequences | Frequency(%) |
| --- | --- | --- | --- | --- |
| GF1-SP | BV04-01 | BJ05-01 | CASSPGTGGYEQYF | 0.19 |
|  | BV13-01,  13-03 | BJ02-07 | CASSDRDSYEQYF | 0.1 |
|  | BV13-02,  13-03 | BJ01-02 | CASSSGFSDYTF | 0.09 |
|  | BV13-01,  13-02,13-03 | BJ02-01 | CASGDTYAEQFF | 0.07 |
|  | BV13-01,  13-02 | BJ02-01 | CASSLGRGAEQFF | 0.07 |
| GF2-In | BV04-01 | BJ02-01 | CASSRDWDYAEQFF | 5.08 |
|  | BV13-01 | BJ01-04 | CASSDGGRNSNERLFF | 3.39 |
|  | BV13-01 | BJ01-02 | CASSDGQGDSDYTF | 3.39 |
|  | BV13-01 | BJ02-07 | CASSEDFYEQYF | 3.39 |
|  | BV03-01 | BJ02-07 | CASSPDRASYEQYF | 3.39 |
| GF2-SP | BV13-01 | BJ02-07 | CASSHWGSSYEQYF | 1.0 |
|  | BV31-01 | BJ02-07 | CAWSPGHYEQYF | 0.46 |
|  | BV02-01 | BJ01-03 | CASSHGQALSGNTLYF | 0.36 |
|  | BV04-01 | BJ02-07 | CASSPWGGGYEQYF | 0.36 |
|  | BV19-01 | BJ01-03 | CASSFSGNTLYF | 0.31 |
| GF3-In | BV01-01 | BJ02-07 | CTCSADGQGEQYF | 2.44 |
|  | BV01-01 | BJ02-07 | CTCSAGDNSYEQYF | 2.44 |
|  | BV24-01 | BJ02-07 | CAIRDWGGFEQYF | 1.63 |
|  | BV13-02 | BJ02-07 | CASGDADTYEQYF | 1.63 |
|  | BV13-02 | BJ02-01 | CASGDEDWGGYAEQFF | 1.63 |
| GF3-SP | BV19-01 | BJ02-07 | CASSMGGSYEQYF | 0.1 |
|  | BV20-01 | BJ02-07 | CGAREGSSYEQYF | 0.09 |
|  | BV19-01 | BJ01-04 | CASRWGQSNERLFF | 0.09 |
|  | BV29-01 | BJ01-01 | CASSLGTGNTEVFF | 0.08 |
|  | BV1901 | BJ02-07 | CASSPRALEQYF | 0.08 |

GF: Germ-free class mice; SP: spleen In: intestine.

Table 3 the top five expressed CDR3 sequences from the SPF group of BALB/c mice in spleen and intestine

| SampLe | V gene | J gene | CDR3 sequences | Frequency(%) |
| --- | --- | --- | --- | --- |
| SPF1-In | BV13-01 | BJ02-01 | CARDWGGGAEQFF | 3.89 |
|  | BV13-01 | BJ01-03 | CASSDGGSGNTLYF | 3.89 |
|  | BV02-01 | BJ01-05 | CASSQDVNQAPLF | 3.89 |
|  | BV13-02 | BJ02-07 | CASGEQGFEQYF | 2.59 |
|  | BV13-03 | BJ01-02 | CASKTGGRTNSDYTF | 2.59 |
| SPF1-SP | BV19-01 | BJ02-02 | CASRNPGQKNTGQLYF | 0.45 |
|  | BV13-01,  13-02 | BJ01-03 | CASSDGGSGNTLYF | 0.19 |
|  | BV05-01 | BJ02-07 | CASSQEETGGYEQYF | 0.19 |
|  | BV14-01 | BJ01-02 | CASSFGTGDSDYTF | 0.18 |
|  | BV05-01 | BJ02-05 | CASSQDWGIDTQYF | 0.18 |
| SPF2-In | BV31-01 | BJ02-01 | CAWGRLGGSEQFF | 11.11 |
|  | BV05-01 | BJ02-07 | CASSQDIYEQYF | 8.33 |
|  | BV19-01 | BJ02-07 | CASENRGLSYEQYF | 5.55 |
|  | BV13-03 | BJ01-04 | CASSVGTKSNERLFF | 5.55 |
|  | BV18-01 | BJ02-02 | WSPIANTGQLYF | 5.55 |
| SPF2-SP | BV13-01,  13-02,13-03 | BJ01-05 | CASKSGNNNQAPLF | 0.27 |
|  | BV19-01 | BJ01-05 | CASRQNNNQAPLF | 0.21 |
|  | BV19-01 | BJ02-07 | CASSRGDRGSYEQYF | 0.16 |
|  | BV19-01 | BJ02-07 | CASSYSSYEQYF | 0.13 |
|  | BV19-01 | BJ01-05 | CASKQNNNQAPLF | 0.13 |
| SPF3-In | BV19-01 | BJ02-07 | CASSIQGLGSYEQYF | 5.19 |
|  | BV04-01 | BJ02-07 | CASSYIPYEQYF | 4.48 |
|  | BV01-01 | BJ01-03 | CTCSASGSGNTLYF | 2.78 |
|  | BV01-01 | BJ02-03 | CTCSADSAETLYF | 1.97 |
|  | BV13-01 | BJ01-05 | CASSDGTGGNQAPLF | 1.7 |

SPF: Specific Pathogen-free mice; SP: spleen In: intestine.

Table 4 the top five expressed CDR3 sequences from the CL group of BALB/c mice in spleen and intestine

| SampLe | V gene | J gene | CDR3 sequences | Frequency(%) |
| --- | --- | --- | --- | --- |
| CL1-In | BV04-01 | BJ02-07 | CASSFGQSSYEQYF | 2.01 |
|  | BV16-01 | BJ02-07 | CASSLVHYEQYF | 2.01 |
|  | BV19-01 | BJ01-02 | CASSMYNSGNTLYF | 2.01 |
|  | BV05-01 | BJ02-03 | CASSQDRRGSAETLYF | 2.01 |
|  | BV02-01 | BJ01-03 | CASSQEQGNSGNTLYF | 2.01 |
| CL1-SP | BV13-03 | BJ01-03 | CASSGTSGNTLYF | 0.19 |
|  | BV05-01 | BJ02-07 | CASSHPGLGKYEQYF | 0.18 |
|  | BV19-01 | BJ02-07 | CASSRTGGKGEQYF | 0.16 |
|  | BV01-01 | BJ02-07 | CTCSAETGGYEQYF | 0.15 |
|  | BV05-01 | BJ01-03 | CASSQVGTGNTLYF | 0.14 |
| CL2-In | BV19-01 | BJ02-07 | CASRTGPYEQYF | 4.41 |
|  | BV20-01 | BJ01-04 | CGAREGSNERLFF | 4.41 |
|  | BV13-02 | BJ01-02 | CASGDGGANSDYTF | 2.94 |
|  | BV13-02 | BJ01-05 | CASGSRDNNQAPLF | 2.94 |
|  | BV13-01 | BJ01-04 | CASSDPGPNERLFF | 2.94 |
| CL2-SP | BV01-01 | BJ02-07 | CTCSGGHHEQYF | 0.15 |
|  | BV04-01 | BJ01-05 | CASSPTGPAPLF | 0.12 |
|  | BV13-01,  13-02 | BJ01-05 | CASSGNNQAPLF | 0.11 |
|  | BV19-01,  02-01 | BJ02-07 | CASSRDRYEQYF | 0.08 |
|  | BV04-01 | BJ02-07 | CASSFSYEQYF | 0.09 |
| CL3-In | BV13-02 | BJ02-07 | CASGGDNEQYF | 10 |
|  | BV15-01 | BJ02-07 | CASRTGGYEQYF | 10 |
|  | BV30-01 | BJ02-04 | CSSRTGGGQNTLYF | 10 |
|  | BV27-01 | BJ01-03 | RDASCVSGNTLYF | 10 |
|  | BV04-01 | BJ02-04 | CASSFGTTSQNTLYF | 5 |
| CL3-SP | BV13-02,  13-03 | BJ02-07 | CASRTGQIYEQYF | 0.2 |
|  | BV19-01 | BJ02-07 | CASSIREGEQYF | 0.19 |
|  | BV01-01 | BJ01-05 | CTCSADTGGQAPLF | 0.14 |
|  | BV02-01 | BJ01-03 | CASSQEFLSGNTLYF | 0.13 |
|  | BV26-01 | BJ02-07 | CASSLGGTGDHEQYF | 0.12 |

CL: Clean Class mice; SP: spleen In: intestine.

**Additional file 4:**


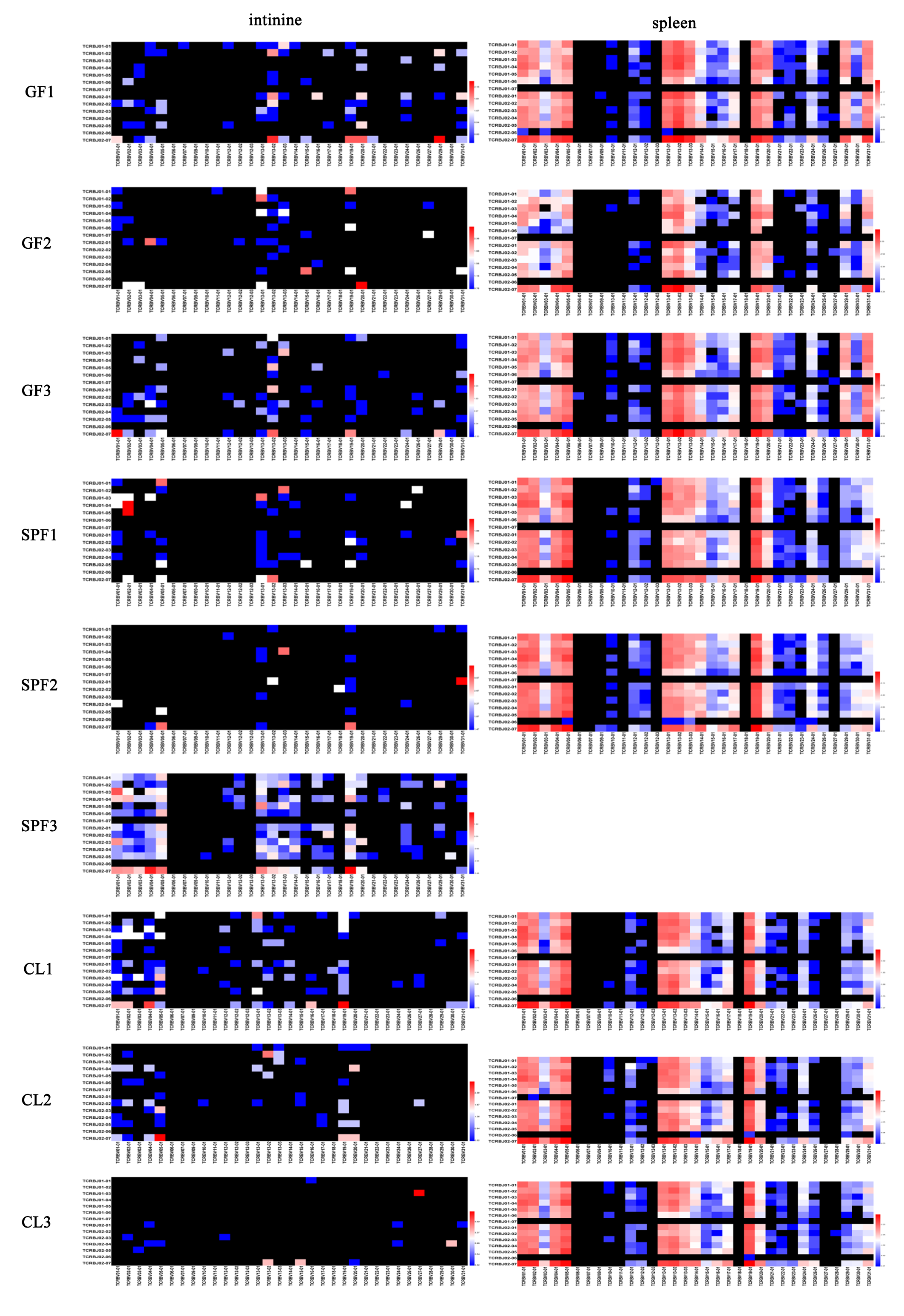


Figure 3 Usage frequency of *TRBV*-*TRBJ* combinations gene segments in spleen and intestinal from the 9 mice. GF: Germ-free class mice; SPF: Specific Pathogen-free mice; CL: Clean Class mice.
